# Effects of aging on serum levels of lipid molecular species as determined by lipidomics analysis in Japanese men and women

**DOI:** 10.1186/s12944-018-0785-6

**Published:** 2018-06-06

**Authors:** Noriaki Kawanishi, Yuki Kato, Kyosuke Yokozeki, Shuji Sawada, Ryota Sakurai, Yoshinori Fujiwara, Shoji Shinkai, Nobuhito Goda, Katsuhiko Suzuki

**Affiliations:** 1 Institute for Nanoscience & Nanotechnology, Waseda University, 513, Tsurunomaki, Waseda, Shinjuku-ku, Tokyo, 162-0041 Japan; 2 Chiba Institute of Technology, Faculty of Advanced Engineering, Narashino, Chiba, Japan; 3 Guraduate School of Health and Sports Science, Juntendo University, Inzai, Chiba, Japan; 4 Department of Life Science and Medical Bioscience, School of Advanced Science and Engineering, Waseda University, Shinjuku-ku, Tokyo, Japan; 5 Faculty of Sports Science, Waseda University, Tokorozawa, Saitama, Japan; 6 Research Team for Social Participation and Community Health, Tokyo Metropolitan Institute of Gerontology, Itabashi-ku, Tokyo, Japan

**Keywords:** Aging, Lipidomics, Lipid, Molecular species

## Abstract

**Background:**

Aging is known to be associated with increased risk of lipid disorders related to the development of type 2 diabetes. Recent evidence revealed that change of lipid molecule species in blood is associated with the risk of type 2 diabetes. However, changes in lipid molecular species induced by aging are still unknown.

We assessed the effects of age on the serum levels of lipid molecular species as determined by lipidomics analysis.

**Methods:**

Serum samples were collected from ten elderly men (71.7 ± 0.5 years old) and women (70.2 ± 1.0 years old), ten young men (23.9 ± 0.4 years old), and women (23.9 ± 0.7 years old). Serum levels of lipid molecular species were determined by liquid chromatography mass spectrometry-based lipidomics analysis.

**Results:**

Our mass spectrometry analysis revealed increases in the levels of multiple triacylglycerol molecular species in the serum of elderly men and women. Moreover, serum levels of total ester-linked phosphatidylcholine (PC) and phosphatidylethanolamine (PE) were increased by aging. In contrast, serum levels of specific ether-linked PC and PE molecular species were lower in elderly individuals than in young individuals.

**Conclusions:**

Our finding indicates that specific lipid molecular species, such as ether- and ester- linked phospholipids, may be selectively altered by aging.

## Background

Aging is known to cause the development of metabolic syndrome, including type 2 diabetes and atherosclerosis [1]. Patients with metabolic syndrome have a unique type of dyslipidemia characterized by hypertriglyceridemia and hypercholesterolemia [2]. Thus, information regarding abnormal levels of triacylglycerol (TAG) and cholesterol in the blood is generally used to evaluate the risk for metabolic syndrome. Importantly, aging is known to be associated with increased risk of lipid disorders related to the development of metabolic syndrome [3]. Lipids have many key biological functions and act as structural components of cell membranes, energy storage sources, and mediators of signaling pathways [4]. On the basis of their structures and functions, lipids can be categorized into distinct classes, such as free fatty acids, glycerolipids, and glycerophospholipids.

Additionally, each lipid class has distinct molecular species based on structure. For example, glycerolipids, such as TAG and diacylglycerol (DAG), are composed of glycerol and fatty acids, and individual molecular species of glycerolipids have distinct effects on biological functions. Interestingly, TAGs containing saturated fatty acids (e.g., palmitate) impair the insulin signaling pathway in the skeletal muscle [5]. Recent studies using comprehensive analytical methods for lipid profiling have revealed that some lipid molecules in the plasma and serum are associated with the risk of metabolic syndrome [6–10]. For example, high plasma levels of specific TAG molecular species (e.g., TAG 50:0 and TAG 52:1) have been observed in patients with type 2 diabetes [6]. These findings suggest that the level of specific lipid molecular species may be beneficial as a biomarker for assessing the pathological status of metabolic syndrome.

In addition to TAG, ether- and ester-linked phospholipids have been examined in the relation to metabolic syndrome and cardiovascular disease. Although phospholipids can be classified into ester-linked and ether-linked phospholipids, ester-linked phospholipids are characterized by the glycerol backbone sn-1 and sn-2 positions have acyl chains attached by ester bonds. On the other hand, ether-linked phospholipids are characterized by an ether linkage between the glycerol backbone and one or both fatty acid side chains (usually the sn-1 position). Ether-linked lipids may function as free radical scavengers and thereby exhibit protective effects on cells and tissues by mitigating oxidative stress. Importantly, recent studies have shown that plasma levels of specific ester-linked phosphatidylcholine (PC) molecular species (e.g., PC 34:2 and PC 36:2) are increased in patients with type 2 diabetes [6, 7]. In contrast, plasma levels of specific ether-linked PC molecular species are decreased in patients with type 2 diabetes [8]. Those findings indicate that ester-linked lipid and ether-linked lipid exert distinct effects on development of type 2 diabetes. Therefore, it is important to examine the effects of aging on ester- and ether-linked lipids.

Aging is known to increase the risk of metabolic syndrome via the development of dyslipidemia [2]. Previous studies have shown that plasma levels of total triglycerides and cholesterol are higher in elderly participants than in the young ones. However, changes in lipid molecular species induced by aging are still unknown. Here, we used lipidomics analysis to investigate lipid molecular species profiles associated with aging. We assessed the effects of age on the serum levels of aging-associated lipid molecular species in men and women.

## Methods

### Participants

Elderly individuals aged 66–74 years were recruited from participants who qualified during specific health checkups conducted at Kusatsu-machi, Gunma Prefecture, Japan, with the cooperation of the Tokyo Metropolitan Institute of Gerontology. Additionally, young individuals aged 21–27 years were recruited from Waseda University. Data from 10 elderly men and women and 10 young men and women were used in this study. Table 1 shows the age, height, weight, and body mass index (BMI) of each participant.

**Table 1:** Characteristics in young subject and older participants

	Age	Height	Weight	BMI
Men				
Young	23.9 ± 0.7	172.6 ± 1.7	66.7 ± 2.1	22.3 ± 0.4
Elderly	71.7 ± 0.5^a^	163.8 ± 1.9^a^	68.2 ± 2.5	25.4 ± 0.8
Women				
Young	23.9 ± 0.4	160.8 ± 1.5	52.2 ± 2.6	20.2 ± 0.9
Elderly	70.2 ± 1.0^a^	148.2 ± 2.2^a^	50.4 ± 3.8	23.0 ± 0.7^a^

^a^Significantly different between young participants and elder participants (Unpaired Student’s t test, *P* < 0.05)

Written informed consent was obtained from all participants. Ethical approval for this study conformed to the standards of the Declaration of Helsinki. The study protocol was approved by the ethics committees of the Tokyo Metropolitan Institute of Gerontology and Waseda University.

### Blood sampling

Venous blood samples were obtained from the forearms of participants at rest. Serum samples were prepared by centrifugation of the whole blood at 1000×*g* for 10 min after incubation for 30 min at room temperature.

### Lipidomics analysis

To examine the serum lipid profiles, liquid chromatography mass spectrometry (LC/MS)-based lipidomics analysis was performed. Lipid fractions of the serum samples (50uL) were extracted using 750 μl of methanol/chloroform (2:1) containing a 1 ppm internal standard (17:0–17:1–17:0 d5-TAG (Avanti Polar Lipids, Inc., Alabama, USA) and Octadecanoic-d35 acid (Larodan, Solna, Sweden) for positive and negative ion mode analysis, respectively). Lipid extracts were analyzed on an AB Sciex TripleTOF 4600 mass spectrometer combined with liquid chromatography (Shimadzu 20A, Shimadzu Corp., Kyoto, Japan) using a column for analysis (HSS-C18 2.11 × 50 mm 1.7 mm; Nihon Waters K.K., Tokyo, Japan). The binary solvent solutions were used as follows: solvent A containing methanol/acetonitrile/water (19:19:2) and solvent B containing isopropanol containing 0.1% acetic acid and 0.028% ammonia for positive ion mode. Solvent A containing 60% acetonitrile in water and solvent B containing 50% acetonitrile and 50% isopropanol containing 0.1% acetic acid and 0.028% ammonia for negative ion mode. Mass spectrometry was performed in the negative-ion mode for free fatty acids (FFA), PC, PE, and SM, and in positive-ion mode for TAG. Data were collected at a mass range of *m/z* (120–1200) and processed using software. Lipids were identified using an internal spectral library or with mass spectrometry.

### Statistical analysis

Previous evidence indicates that there is difference in triacylglycerol levels in blood between men and women, because sex hormone such as estrogen affect lipid metabolism [11]. For these reasons, men and women were compared separately. Serum levels of lipid molecular species and participant characteristics parameter (age, height, weight, and BMI) in the young and elderly participants were compared by Student’s unpaired *t*-tests. Differences with *P* values of less than 0.05 were considered significant.

## Results

### Participant characteristics

There were differences in the average age and BMI between the young and elderly participants. In men and women, the BMI was significantly higher in elderly participants than in young participants (Table 1).

### Lipidomics analysis

**Fig. 1:**
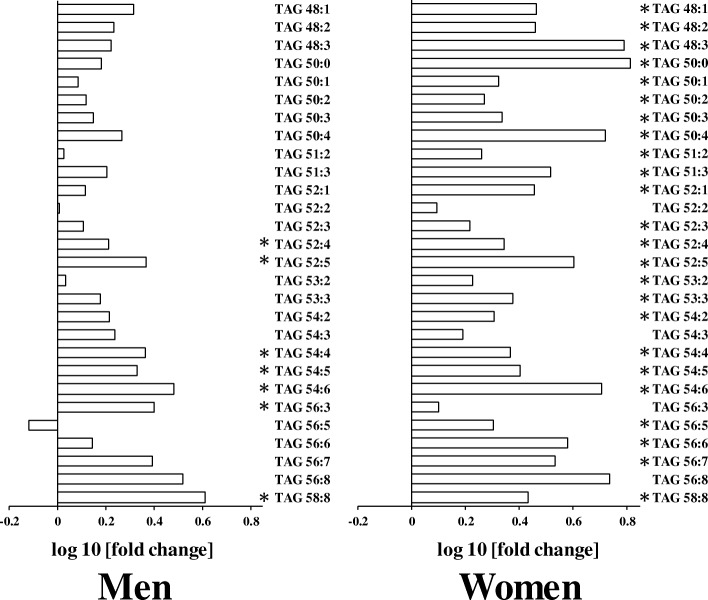
Differences in serum levels of triacylglycerol (TAG) molecular species in young and elderly (**a**) men and (**b**) women. The log10 fold changes in TAG molecular species are shown. *, *P* < 0.05. Positive values indicate lipid level higher in elderly. Negative values indicate lipid level higher in young

We performed lipidomics analysis by mass spectrometry to evaluate lipid molecular species and FFA in serum. As a result, we identified four lipid classes. A comparison of serum TAG levels between the young and elderly participants is shown in Fig. 1. Our mass spectrometry analysis revealed increases in the levels of multiple TAG molecular species in the serum of elderly men and women (Fig. 1). Serum levels of specific TAG molecular species were higher in elderly individuals than in young individuals. Differences in serum levels of specific TAG molecular species between the elderly and young individuals were observed in seven species in men and 24 species in women.

**Fig. 2:**
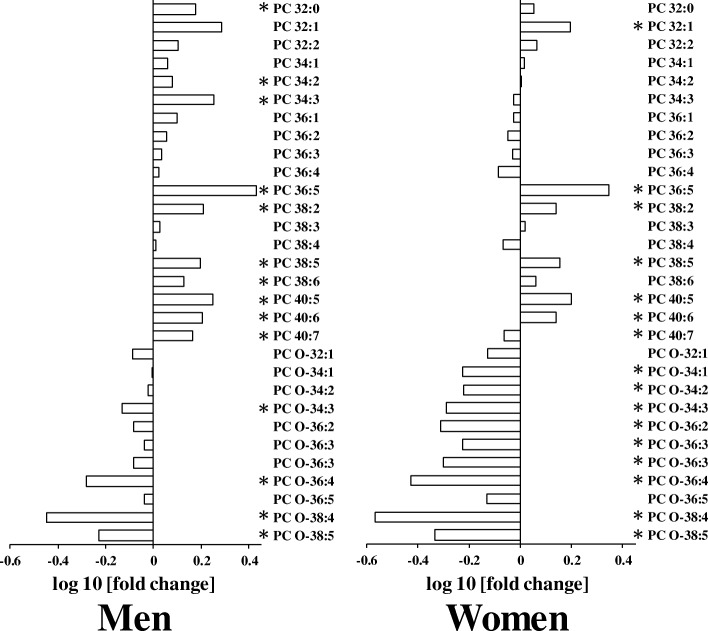
Differences in serum levels of phosphatidylcholine (PC) molecular species in young and elderly (**a**) men and (**b**) women. The log10 fold changes in TAG molecular species are shown. *, *P* < 0.05. Positive values indicate lipid level higher in elderly. Negative values indicate lipid level higher in young

**Fig. 3:**
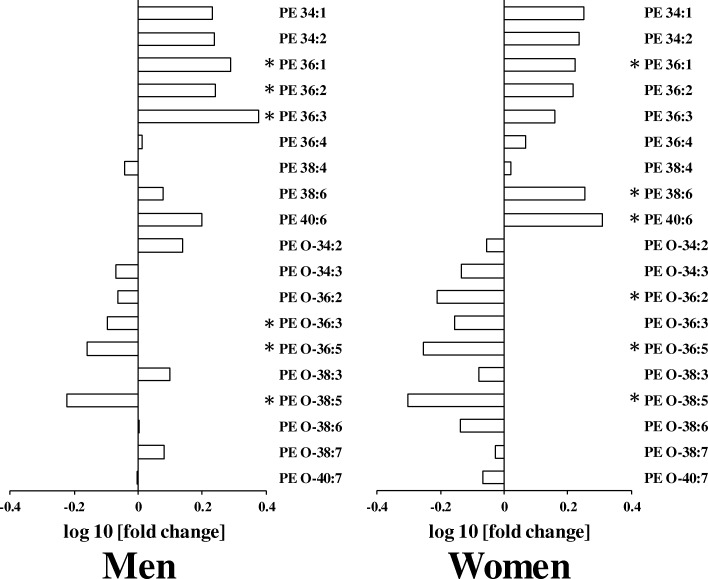
Differences in serum levels of phosphatidylethanolamine (PE) molecular species in young and elderly (**a**) men and (**b**) women. The log10 fold changes in TAG molecular species are shown. *, *P* < 0.05. Positive values indicate lipid level higher in elderly. Negative values indicate lipid level higher in young

We also examined whether serum phospholipids were affected by aging. Similar to TAG levels, serum levels of total ester-linked PC and phosphatidylethanolamine (PE) were increased by aging (Figs. 2 and 3). However, variations in ether-linked PC and PE molecular species were different from those in ester-linked PC and PE. Importantly, we found that serum levels of specific ether-linked PC and PE molecular species (e.g., PC O-36:4 and PE O-36:5) were lower in elderly individuals than in young individuals (Figs. 2 and 3).

**Fig. 4:**
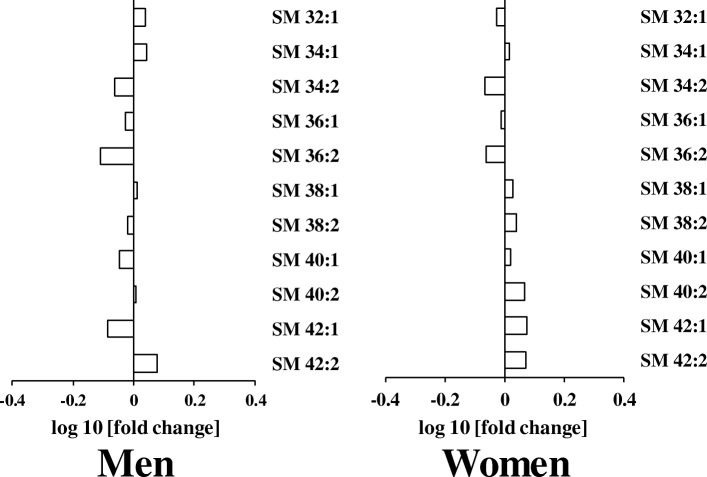
Differences in serum levels of sphingomyelin (SM) molecular species in young and elderly (**a**) men and (**b**) women. The log10 fold changes in TAG molecular species are shown. *, *P* < 0.05. Positive values indicate lipid level higher in elderly. Negative values indicate lipid level higher in young

We further studied whether serum levels of sphingomyelin (SM) molecular species differed with aging. Our results showed that there were no differences in SM molecular species levels between the elderly and young participants (Fig. 4).

**Fig. 5:**
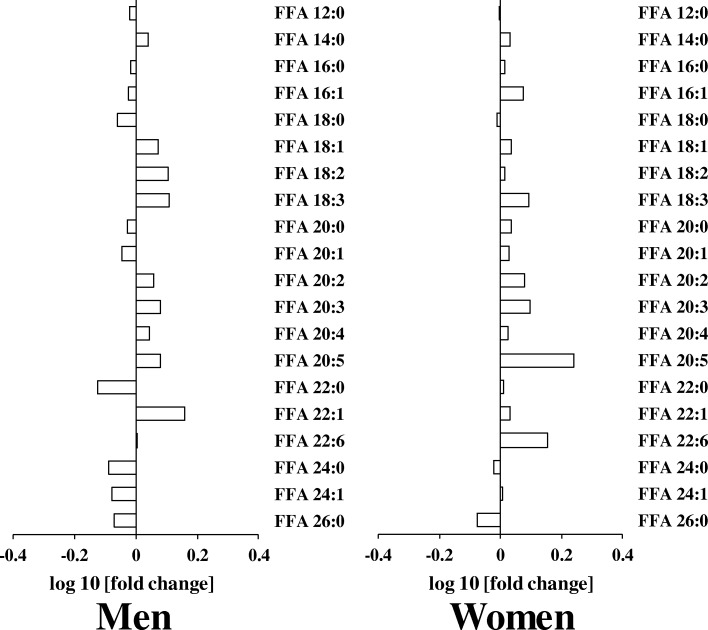
Differences in serum levels of free fatty acid (FFA)s in young and elderly (**a**) men and (**b**) women. The log10 fold changes in FFAs are shown. *, *P* < 0.05. Positive values indicate lipid level higher in elderly. Negative values indicate lipid level higher in young

We also examined whether serum FFA were affected by aging. Although we identified 20 FFA including long chain fatty acids (carbon chain length ≥ 12), serum levels of these FFA were not different between the elderly and young participants (Fig. 5).

## Discussion

The roles of lipids in the development of chronic disease have been assessed in many previous studies [2]. In fact, lipid parameters such as FFA, TAG and SM are used as biomarkers for age-related diseases, including type 2 diabetes and cardiovascular disease [12, 13]. Plasm and serum levels of FFAs are known to increase in obese patients and contribute to type 2 diabetes and several cardiovascular diseases. Importantly, higher levels of saturated fatty acids such as palmitate impair insulin signaling in skeletal muscle by induction of systemic lipotoxicity, with subsequent development of type 2 diabetes [14]. In addition, higher levels of polyunsaturated omega-6 fatty acids such as arachidonic acid may cause myocardial injury by induction of systemic inflammation, with subsequent development of cardiovascular disease [15]. This evidence suggests that elevation of specific free fatty acids may play important roles in type 2 diabetes and cardiovascular diseases. However, in our study, mass spectrometry analysis showed there is no difference in the long-chain FFA including palmitate and arachidonic acid levels of healthy elderly people and young people. Interestingly, recent evidence has also revealed specific lipid molecules species associated with the risk of several diseases, such as type 2 diabetes, atherosclerosis, and cardiovascular disease [6–10]. Importantly, plasma or serum levels of specific TAG, PC, and SM molecular species are increased or decreased in patients with type 2 diabetes. Therefore, these lipid molecular species are thought to be beneficial as biomarkers for assessing the pathological status of type 2 diabetes. Aging is known to cause dyslipidemia, e.g., increased levels of total TAG and cholesterol in the blood [3]; however, the mechanisms associated with these aging-induced changes in lipid molecular species are not well understood. In this study, we investigated whether these lipid parameters were beneficial as biomarkers of aging by using lipidomics analysis to evaluate the aging-induced changes in the serum levels of lipid molecular species.

In this study, we identified the profiles of four lipid classes, TAG, PC, PE, and SM. Although TAGs containing saturated fatty acids undergo β-oxidation in the mitochondria, aging induces impaired activity of β-oxidation enzymes [16]. Palmitate is among these TAG molecular species and is known to cause glucose metabolic disorder by the impairment of insulin signaling in skeletal muscle cells and adipocytes [5]. A recent study reported that high levels of specific TAG molecular species, such as TAG 48:1, 50:0, and 52:1, are associated with an increased risk of type 2 diabetes [6]. Interestingly, we found that serum levels of these TAG molecular species (TAG 48:1, 50:0, and 52:1) were higher in elderly individuals than in young individuals. Importantly, several studies reported that high levels of specific TAG molecular species containing long-chain fatty acids are associated with an increased risk of cardiovascular disease [17, 18]. In this study, we observed that serum levels of most TAG molecular species containing long-chain fatty acids differed between the elderly and young participants. These results indicate that increased blood levels of specific TAG molecular species, including saturated fatty acids such as palmitate, with aging may be associated with increased risk of type 2 diabetes and cardiovascular disease with aging.

Glycerophospholipids, such as PC and PE, are structural components of the cell membrane. Importantly, phospholipids can be classified into ester-linked and ether-linked phospholipids. Ether-linked phospholipids have antioxidant activity and thereby exhibit protective effects on cells and tissues by mitigating oxidative stress [19]. Interestingly, low serum levels of ether-linked PC are associated with an increased risk of type 2 diabetes and hypertension [9, 20]. Moreover, Meikle et al. reported that plasma levels of ether-linked PC are decreased in patients with cardiovascular disease. In this study, we found that aging induced alterations in the patterns of PC and PE molecular species. For example, we observed that some molecular species of ester-linked phospholipids were higher in elderly participants than in young participants. In contrast to ester-linked phospholipids, serum levels of ether-linked PC and PE molecular species (e.g., PC O-36:4 and PE O-36:5) were lower in elderly participants than in young participants. Differences in serum ether-linked phospholipid levels between the elderly and young individuals were observed in six men (four for PC and three for PE) and 12 women (nine for PC and three for PE). Taken together, our data reveal that the patterns of PC and PE molecular species differ among the elderly and young individuals. Thus, lower levels of ether-linked phospholipids in the elderly may reflect impaired antioxidant capacity and disease risk such as type 2 diabetes and cardiovascular disease.

SM is a structural component of the cell membrane and has a role as a cellular messenger [21]. Interestingly, low levels of specific SM molecular species are linked to an increased risk of type 2 diabetes, cardiovascular disease and neurodegenerative disease [22, 23]. In fact, Han et al. [24] reported that plasma levels of SM containing long-chain fatty acid are decreased in patients with Alzheimer’s disease. Furthermore, low levels of specific SM molecular species (e.g., SM 32:1) have been observed in patients with type 2 diabetes [8, 25]. Thus, characterization of SM molecular species has highlighted specific lipids underlying aging-related neurodegenerative and metabolic diseases. In this study, we found that there were no differences in the serum levels of SM molecular species between the young and elderly participants. Thus, these results suggest that the levels of SM molecular species in the serum may not be affected by age.

A limitation of this study was that it did not reveal whether aging caused changes in fatty acid composition in lipid class. Recent evidence indicates that alterations in the fatty acid composition of lipids may be an important factor that modulates physiological function such as insulin action [5, 26]. Future studies should investigate changes in fatty acid composition by aging using tandem mass spectrometry (MS/MS) platform for identifying incorporated fatty acids in lipid class.

There were significant differences in the lipid profiles between young and elderly participants. However, the mechanisms through which lipid molecular species patterns are altered by aging are still unclear. Interestingly, [10] reported that plasma levels of multiple TAG molecular species are higher in obese individuals. In contrast, the ratio of ether-linked phospholipids to ester-linked phospholipids is lower in obese individuals [10]. Another study has also shown that serum levels of ether-linked phospholipids are decreased in obese individuals compared with those in lean participants [9]. Moreover, Colas et al. [20] found that the ratio of ether-linked phospholipids to ester-linked phospholipids is negatively correlated with waist circumference. These data indicate that lipid profiles may be altered by obesity. In this study, the elderly participants had significantly higher BMIs than young participants of either sex. Importantly, we also found that the levels of total ether-linked PC in the serum of elderly were negatively correlated with the BMI (*r* = − 0.349, *P* < 0.05, data not shown). In contrast, we also observed that the levels of total ester-linked PC in the serum of elderly were correlated with the BMI (*r* = 0.337, *P* < 0.05, data not shown). These results suggest that differences in BMIs between the young and elderly participants may affect lipid profiles, including lipid molecular species patterns.

Although physical activity decreases with age, the risk of dyslipidemia in relation to the level of physical activity is known to increased [27]. In fact, a previous report demonstrated that the amount of physical activity in older individuals is negatively correlated with the BMI and blood levels of triglycerides and cholesterol [28]. Thus, these studies suggest that increased levels of physical activity may improve lipid profiles; however, the effects of physical activity on lipid molecular species patterns are unknown. Additional studies are required to elucidate the combined or independent effects of physical activity on decreasing BMIs and on changes in lipid profiles, including alterations in lipid molecular species patterns.

## Conclusion

Our results demonstrate the effects of aging on lipid profiles in the blood. Importantly, our study shows that specific lipid molecular species, such as ether-linked phospholipids, may be selectively altered by aging. Our findings suggest that these lipid molecular species may be used as indicators of aging.
